# Having it both ways: how STAT3 deficiency blocks graft-versus-host disease while preserving graft-versus-leukemia activity

**DOI:** 10.1172/JCI172251

**Published:** 2023-08-01

**Authors:** Joshua D. Brandstadter, Riley Outen, Ivan Maillard

**Affiliations:** Division of Hematology/Oncology, Perelman School of Medicine, University of Pennsylvania, Philadelphia, Pennsylvania, USA.

## Abstract

Allogeneic hematopoietic cell transplantation can cure patients with high-risk leukemia through graft-versus-leukemia (GVL) effects, the process by which malignant leukemic cells are cleared by donor-derived immune cells from the graft. The problem of harnessing GVL effects while controlling inflammation and host-organ damage linked with graft-versus-host disease (GVHD) has been the most formidable hurdle facing allogeneic hematopoietic cell transplantation. This powerful, curative-intent therapy remains among the most toxic treatments in the hematologist’s armamentarium due to the combined risks of GVHD-related morbidity, infections, and leukemia relapse. In this issue of the *JCI*, Li, Wang, et al. report that T cell *Stat3* deficiency can extricate GVL effects from GVHD through tissue-specific programmed death-ligand 1/programmed cell death protein 1–dependent (PD-L1/PD-1-dependent) bioenergetic alterations that blunt harmful T cell effects in GVHD target organs, while preserving their beneficial antitumor activity in lymphohematopoietic tissues.

## In search of the transplantation holy grail

In patients with blood cancers, the goal of allogeneic hematopoietic cell transplantation is to unleash curative graft-versus-leukemia (GVL) effects while minimizing morbidity from graft-versus-host disease (GVHD) (1). It has been challenging to disentangle the benefits of GVL from the harms of GVHD, as both are mediated by alloreactive donor T cells recognizing foreign tissue antigens. Patients receiving autologous grafts or T cell–depleted allogeneic transplants have lower GVHD rates, but higher risks of relapse. Patients who receive donor lymphocyte infusions can achieve remission of recurrent disease, but with a risk of enhanced GVHD. Current GVHD prophylactic strategies involve global immunosuppression whereby reduced GVHD severity in target tissues (e.g., gut, liver, skin, and lung) is achieved at the increased cost of leukemia relapse and impaired antiinfective immunity.

The STAT3 pathway relays signals from multiple cytokines, including IL-6, IL-17, IL-21, and IL-23, with an impact on T cell differentiation and autoimmune disorders, making it an ideal candidate for a role in alloimmunity (2). Early research on STAT3 found reduced GVHD-related mortality in mice treated with an inhibitor of STAT3 phosphorylation (2). *Stat3* inactivation in CD4^+^ T cells prevented chronic sclerodermatous GVHD in mice and limited donor T cell accumulation in vivo while expanding Tregs and decreasing numbers of inflammatory Th17 cells (3). In other work, *Stat3* deletion preserved natural Tregs and expanded induced Tregs (iTregs) to protect from acute GVHD (4). Pharmacological inhibition of phosphorylated STAT3 in human Tregs also prevented skin graft rejection in immunodeficient mice and reduced xenogeneic GVHD (5).

**Figure 1:**
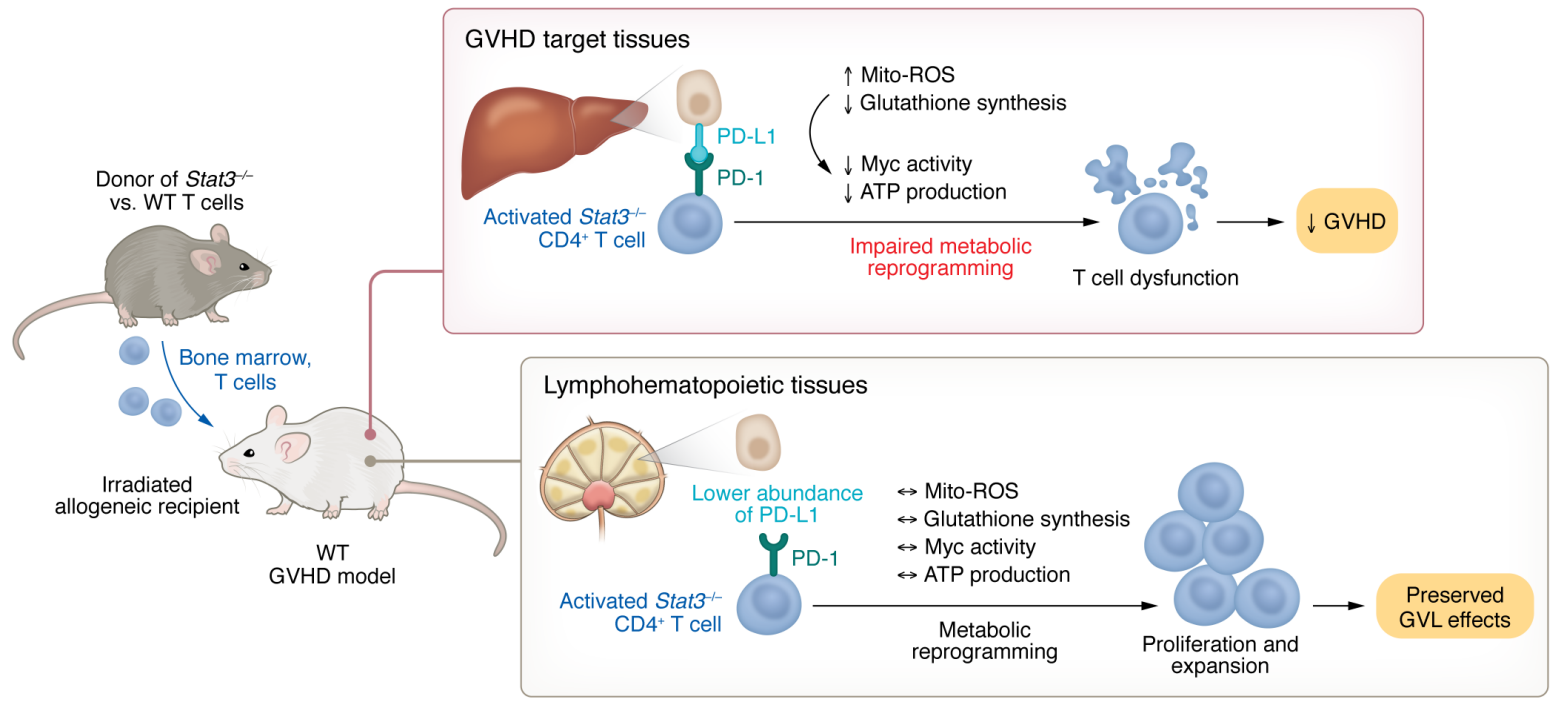
STAT3 deficiency prevents GVHD in target organs while preserving GVL activity in lymphohematopoietic tissues. In GVHD target organs, activated T cells undergo metabolic reprogramming to sustain inflammatory T cell responses via the glutathione pathway. However, in the absence of STAT3, PD-1 signaling triggers high levels of Mito-ROS and decreased glutathione synthesis, which inhibits Myc activity and ATP production to impair metabolic reprogramming. These changes lead to T cell dysfunction, limiting T cell expansion and effector functions in target tissues, thus preventing GVHD. The lower abundance of PD-L1 in the spleen and other lymphohematopoietic tissues leads to lower PD-1 signaling, which allows metabolic reprogramming that supports T cell expansion and preserves GVL activity.

In this issue of the *JCI*, Li, Wang, et al. describe how *Stat3* deficiency in donor T cells maintained potent GVL activity while preventing GVHD (6). In a major histocompatibility complex–mismatched mouse model of severe GVHD, the authors demonstrated protection from GVHD with *Stat3*-deficient T cells, but only in the presence of intact programmed death-ligand 1/programmed cell death protein 1 (PD-L1/PD-1) signaling. *Stat3* inactivation in T cells enhanced PD-L1/PD-1–mediated inhibition of metabolic pathways in alloreactive T cells, thus interfering with metabolic reprogramming and leading to T cell dysfunction specifically in GVHD target organs (Figure 1). Because PD-L1 was more abundant in GVHD target tissues compared with lymphohematopoietic organs where hematologic malignancies mostly reside, *Stat3* deficiency had tissue-specific effects that blunted GVHD in target organs, but maintained potent GVL activity. Importantly, studies comparing transplants with low doses of control and *Stat3^–/–^* donor T cells revealed a mild decrease in the GVL effects of *Stat3*-deficient T cells when assessed on a per cell basis. However, recipients tolerated much higher doses of Stat3-deficient T cells due to GVHD protection, thus preserving potent overall GVL activity. Past work from these authors also identified STAT3-dependent expansion and differentiation of pathogenic PSGL^lo^CD4^+^ T cells in the lung and liver of recipients with chronic GVHD (7). Altogether, *Stat3* inactivation had divergent effects at different body sites, thereby controlling GVHD but preserving GVL effects in lymphohematopoietic organs — one potential path toward the transplantation holy grail.

## Target tissue PD-L1 regulates GVHD

Li, Wang, et al. showed that GVHD prevention achieved by *Stat3* loss in T cells required PD-1 signaling in donor T cells mediated by PD-L1 in recipient tissues (6). *Stat3* deficiency reduced glutathione synthesis and increased mitochondrial ROS (mito-ROS) in a PD-1–dependent manner (Figure 1). The antioxidant glutathione pathway is critical for metabolic reprogramming to sustain inflammatory T cell responses after initial T cell activation (8). Consistently, mito-ROS accumulation and decreased glutathione in *Stat3*-deficient alloreactive T cells were associated with decreased Myc expression and activity, decreased ATP production, and evidence of T cell dysfunction in GVHD target tissues (6). Yet it remains unclear where precisely *Stat3* deficiency intersects with PD-1 signaling or its downstream consequences in T cells.

PD-1 blockade can accelerate GVHD in mice and humans (9, 10). The authors from the same research group previously speculated that differential binding of PD-L1 to PD-1 in GVHD target tissues and CD80 in lymphohematopoietic tissues contributed to GVHD protection alongside preservation of GVL (11, 12). Differential expression of PD-L1 in GVHD target organs and lymphohematopoietic tissue was proposed as a mechanism to explain how GVHD was prevented in *Stat3*-deficient T cell recipients, while GVL was largely preserved. Indeed, genetic or pharmacologic inhibition of PD-L1/PD-1 signaling erased GVHD protection from *Stat3* inactivation (6). PD-L1/PD-1 signaling blockade revealed distinct PD-L1/PD-1–dependent alterations in *Stat3*-deficient T cell metabolism between GVHD target organs and lymphohematopoietic tissue. These observations fit with work from others identifying the importance of tissue-resident, locally maintained donor-derived T cells in GVHD (13).

The tissue-specific bioenergetic consequences of *Stat3* deletion should also be considered as part of evolving work on metabolic control of GVHD. While others focused on glycolysis or other bioenergetic pathways in GVHD (14, 15), Li, Wang, et al. reported differential T cell metabolic profiles in spleen and GVHD target organs upon *Stat3* loss (6). Specifically, *Stat3*-deficient T cells showed decreased glutathione/Myc pathway activity and ATP production in the liver, but not in the spleen. These findings should prompt caution in the interpretation of T cell metabolic findings when assessed only in lymphoid organs rather than also in target tissues.

## Tregs or not Tregs

Despite prior findings suggesting a role for Tregs in GVHD protection upon T cell *Stat3* deficiency, Li, Wang, et al. found no impact of Treg depletion on GVHD severity or survival in their model (6). Prior work showed that anti-CD25–mediated Treg depletion reversed protection from *Stat3* deficiency in a mouse model of chronic sclerodermatous GVHD, where anti-CD25 treatment resulted in delayed, organ-specific GVHD in recipients of *Stat3*-deficient T cells (3). Other groups connected Tregs with *Stat3* deficiency’s protection from GVHD based on destabilization of natural Tregs and reduced iTreg differentiation from naive CD4^+^ T cells (4). Finally, human pSTAT3–inhibited iTregs reduced skin graft rejection and protected from xenogeneic GVHD based on a metabolic shift from oxidative phosphorylation to glycolysis, increasing their suppressive function (5). Further work is needed to explain the divergent role reported for Tregs upon STAT3 loss, although unique features of STAT3-targeting methods and individual transplantation models could be at play.

While the Li, Wang, et al. report explored metabolic consequences of *Stat3* deficiency, an important future direction should include identifying key ligands and receptors upstream of STAT3 signaling in GVHD. In past work, these authors speculated that IL-6 and IL-21 could act upstream of STAT3 signaling in pathogenic CXCR5^–^PD-1^hi^ helper T cells implicated in chronic GVHD pathogenesis (16). It is unclear in their current work what might act upstream of STAT3 (6). Preclinical data showed that IL-6 inhibition reduced GVHD while allowing robust GVL, making IL-6 a candidate for acting upstream of STAT3 signaling in this model (17). However, benefits of IL-6 antagonism in GVHD prevention were not substantiated in patients (18). Thus, it is possible that STAT3 integrates IL-6 signaling with other pathways during GVHD pathogenesis.

## STAT3 as therapeutic target for GVHD prophylaxis

Going beyond genetics as a discovery approach, Li, Wang, et al. explored a pharmacological strategy of targeted STAT3 degradation with translational potential (6). They tested a STAT3-selective proteolysis-targeting chimera (PROTAC) degrader, called SD-36, that was developed as an anticancer treatment. SD-36 mediates potent inhibition of signaling by monomeric and dimeric STAT3 without affecting other STAT molecules (19). The STAT3 degrader prevented GVHD when used to pretreat donor T cells ex vivo and also administered to recipients on days 0 and 3 of transplantation, a remarkably short overall treatment duration (6). The high therapeutic activity of transient STAT3 inhibition contrasts with the suggestion that STAT3 activity was essential to sustaining the pathogenic activity of tissue-resident T cells mediating GVHD. However, it is possible that STAT3 activity was particularly relevant for GVHD pathogenesis during seeding of target tissues by T cells early after transplantation. This observation dovetails with findings demonstrating the impact of other transient interventions during the early posttransplant period, such as Notch inhibition, for long-term GVHD control (20). In the future, questions about the feasibility of STAT3 targeting will need to be addressed, including those regarding on-target toxicities of STAT3 inhibition, optimal duration of treatment, and impact on GVL. Concomitant benefits of targeting STAT3 could include direct anticancer effects in STAT3-driven malignancies.

## Conclusions and future directions

T cell *Stat3* deficiency protected mice from GVHD while maintaining potent GVL responses (6). The relative separation between GVHD and GVL achieved by *Stat3* deficiency relied on PD-L1/PD-1 signaling. PD-L1 expression in GVHD target tissues conferred protection from GVHD that was not shared in lymphohematopoietic tissue, where donor T cells are likely to encounter hematological neoplasms. PD-1 signaling in *Stat3*-deficient T cells resulted in inhibition of the glutathione/Myc pathway, culminating in T cell dysfunction. The upstream signals and downstream pathways connecting STAT3 and PD-1 remain subject to further investigation. Furthermore, the role of Tregs appears unresolved given divergent data between Li, Wang, et al. and prior work about the effects of anti-CD25 treatment on GVHD after transplantation with *Stat3*-deficient T cells. Altogether, Li, Wang, et al. propose STAT3 degradation as an attractive strategy for controlling GVHD while maintaining potent GVL activity as well as an interesting concept in which tissue-specific T cell dysfunction explains the distinct effects of STAT3 loss on harmful and beneficial T cell responses (6).

## Version 1 08/01/2023

Electronic publication
